# Endoscopic Verification of Transpapillary Access in Supine Percutaneous Nephrolithotomy: A Prospective Pilot Study Comparing Freehand Ultrasound and Fluoroscopy Guidance

**DOI:** 10.3390/jcm14155604

**Published:** 2025-08-07

**Authors:** Fatih Bicaklioglu, Bilal Eryildirim

**Affiliations:** 1 Department of Urology, Kartal Dr. Lutfi Kirdar City Hospital, 34865 Istanbul, Turkey; bilaleryildirim@yahoo.com; 2 Department of Urology, University of Health Sciences, 34668 Istanbul, Turkey

**Keywords:** percutaneous nephrolithotomy, renal calculi, ultrasonography, fluoroscopy, transpapillary access, nonpapillary access, supine position, ureteroscopy, radiation exposure

## Abstract

**Background/Objectives**: Achieving renal access is a key step in percutaneous nephrolithotomy (PNL), with transpapillary access considered the safest anatomical approach. This prospective pilot study aimed to compare the effectiveness of freehand ultrasound-guided (F-UG) versus fluoroscopy-guided (FG) punctures in achieving anatomically accurate transpapillary access during supine PNL, confirmed by endoscopic visualization. Perioperative and postoperative outcomes were also evaluated. **Methods**: Forty-three patients undergoing supine PNL for renal pelvic or lower calyceal stones were prospectively enrolled and assigned to either the FG group (n = 23) or F-UG group (n = 20). Following renal access, intraoperative flexible ureteroscopy confirmed the anatomical nature of the puncture (transpapillary vs. nonpapillary). The puncture time, fluoroscopy time, operative time, complications (Clavien–Dindo classification), transfusion requirement, hospital stay, and one-month stone-free rates were recorded. **Results**: Transpapillary access was achieved in 95.7% of FG cases and 55.0% of F-UG cases (*p* = 0.003). Radiation exposure was significantly lower in the F-UG group (*p* < 0.001). Complication (15.0% vs. 0.0%) and transfusion rates (10.0% vs. 0.0%) were higher in the F-UG group but not statistically significant (*p* = 0.092 and *p* = 0.210, respectively). Other outcomes, including the operative time, hospital stay, and stone-free rates, were similar between groups. **Conclusions**: FG puncture is more effective for achieving transpapillary access, while F-UG significantly reduces radiation exposure. The endoscopic confirmation method may provide a reference for future comparative studies on access techniques in PNL.

## 1. Introduction

Percutaneous nephrolithotomy (PNL) was first introduced by Fernström and Johansson in 1976 as an alternative to open stone surgery, marking a significant milestone in the management of nephrolithiasis [1]. Over the past five decades, PNL has undergone continuous advancements, incorporating improvements in instrumentation, imaging techniques, and surgical approaches. Despite these developments, it has remained a cornerstone in the surgical treatment of kidney stones, and current urological guidelines continue to recommend PNL as the first-line treatment for renal stones larger than 2 cm [2,3].

The importance of transpapillary access through the calyceal fornix in PNL was highlighted by Sampaio in the early 1990s. His anatomical studies demonstrated that a forniceal puncture minimizes vascular injury compared to an infundibular or pelvic puncture, which risks damaging major renal arteries [4,5]. These findings established the transpapillary approach as a safer and more effective access technique, gaining widespread acceptance until further studies revisited the issue, questioning whether strict adherence to this method was necessary. Liatsikos and colleagues challenged the long-standing preference for forniceal (transpapillary) puncture through a retrospective study of 137 patients, demonstrating that a non-calyceal percutaneous track could be a feasible and safe alternative [6]. Subsequently, a prospective randomized trial further investigated this concept, comparing papillary and infundibular punctures in PNL. The study found no significant difference in bleeding or transfusion rates between the two approaches, raising doubts regarding the necessity of strict adherence to papillary access as the gold standard [7]. Nevertheless, despite these findings, many leading urologists continue to support transpapillary puncture, emphasizing its anatomical advantages in minimizing vascular injury. Alken, one of the pioneers of percutaneous stone surgery, has consistently supported papillary access, citing its safety profile and lower complication rates [8]. Similarly, Pearle argues that while alternative approaches may be feasible, the burden of proof lies with those challenging papillary puncture to demonstrate superior safety and efficacy in large-scale studies [9]. Thus, the debate over the optimal puncture technique remains ongoing, with transpapillary puncture continuing to be widely endorsed in contemporary urological practice.

Regardless of the ongoing discussion on the ideal puncture site, the establishment of precise renal access remains the most critical step in percutaneous nephrolithotomy. Imaging plays a fundamental role in this process, ensuring both accuracy and procedural safety. Fluoroscopy remains the primary and most widely used imaging modality, providing real-time guidance that facilitates accurate needle placement while minimizing complications. In recent years, ultrasound has gained increasing attention as an alternative, particularly for its ability to reduce radiation exposure. While fluoroscopy offers superior visualization of the collecting system, its two-dimensional nature and associated radiation risks are notable limitations. Ultrasound, on the other hand, eliminates radiation exposure and enables direct visualization of renal structures, though it is limited by operator dependency and challenges in obese patients or cases with minimal hydronephrosis [10]. However, while these imaging modalities provide essential guidance for renal access, their ability to accurately confirm transpapillary puncture remains uncertain, posing a significant challenge in standardizing the optimal approach. Several studies have attempted to address this issue by assessing the ability of these imaging modalities to confirm the nature of renal access. Basiri et al. performed ultrasound-guided renal punctures and subsequently verified whether the access was transpapillary using fluoroscopy [11]. Conversely, Tahra et al. conducted a study in which renal access was established under fluoroscopic guidance, and the papillary or nonpapillary nature of the puncture was verified using ultrasound [12]. While fluoroscopy and ultrasound offer valuable guidance for renal access, neither modality provides an absolute method for confirming anatomically accurate transpapillary puncture. The integration of endoscopic combined surgeries has enabled direct visualization of the puncture site within the renal collecting system, revealing that some punctures may inadvertently be nonpapillary despite the surgeon’s intent. Among the available techniques, the most definitive way to verify transpapillary access remains direct endoscopic visualization of the needle puncturing the fornix of the papilla.

In this prospective, comparative pilot study, we aim to compare the outcomes of freehand ultrasound-guided (F-UG) versus fluoroscopy-guided (FG) puncture techniques in achieving transpapillary renal access during supine PNL by directly visualizing the needle puncture site within the collecting system using a flexible ureteroscope. Additionally, perioperative and postoperative parameters were analyzed to assess the efficiency and safety of each technique.

## 2. Materials and Methods

### 2.1. Ethical Approval and Patient Consent

This study was conducted according to the principles of the Declaration of Helsinki. The study protocol was approved by the Institutional Ethics Committee of Kartal Dr. Lutfi Kirdar City Hospital (protocol code: 2023/5l4/24619, approval date: 29 March 2023). Informed consent was obtained from all patients before their inclusion in the study.

### 2.2. Study Design and Patients

This is a prospective, comparative pilot study evaluating the effectiveness of F-UG puncture versus FG puncture for transpapillary access in supine PNL. The study was conducted at Kartal Dr. Lutfi Kirdar City Hospital between February 2023 and January 2025, with 43 patients prospectively enrolled based on predefined criteria.

Inclusion criteria:

- Patients aged 18 years or older;
- Patients with solitary renal pelvis or lower calyceal stone.

Exclusion criteria:

- Patients aged under 18 years;
- Patients with multiple or/and complex stones (partial staghorn, staghorn, etc.);
- Patients with renal anomalies (horseshoe kidney, ectopic kidney, rotation anomaly, etc.);
- Patients with uncontrolled coagulopathy;
- Patients requiring multiple renal accesses (multi-tract PNL);
- Patients in whom flexible ureteroscope could not be advanced to the access site due to stone burden.

### 2.3. Study Outcomes

The primary endpoint of this study was to determine which access technique (F-UG vs. FG) achieves a higher rate of transpapillary access in supine PNL.

Secondary endpoints included perioperative and postoperative parameters to assess the efficiency and safety of each technique. These parameters were the complication rate, hematocrit drop, blood transfusion rate, fluoroscopy time, total operative time, hospitalization time, and stone-free rate.

These parameters were compared between the two groups to evaluate the effectiveness and safety profiles of both access techniques.

### 2.4. Preoperative Evaluation

All patients underwent a standard preoperative evaluation, including laboratory tests such as complete blood count, serum creatinine, liver function tests, coagulation profile, urinalysis, and urine culture. Non-Contrast-Enhanced Computed Tomography (NCCT) was performed on all patients as part of the preoperative imaging assessment. Patients with urinary tract infections in the preoperative period underwent surgery only after receiving appropriate antibiotic treatment.

### 2.5. Surgical Technique

**Figure 1:**
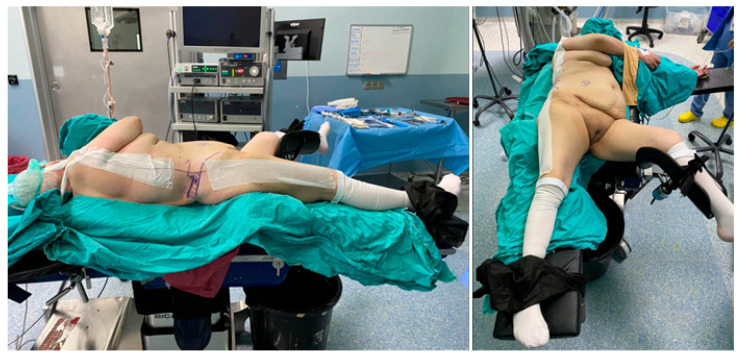
The Barts flank-free supine position.

All PNL procedures were performed in the Barts flank-free supine position [13] (Figure 1). Two surgeons (BE, FB) with expertise in different puncture techniques participated in the study. The surgical procedure began with the placement of a 9.5–11.5 Fr ureteral access sheath (Plastimed^®^, Ankara, Türkiye) over a guidewire (Sensor Guidewire, Boston Scientific^®^, Marlborough, MA, USA) in most patients. In cases where sheath insertion was difficult or not feasible, flexible ureteroscopy was performed directly over the guidewire without using an access sheath.

**Figure 2:**
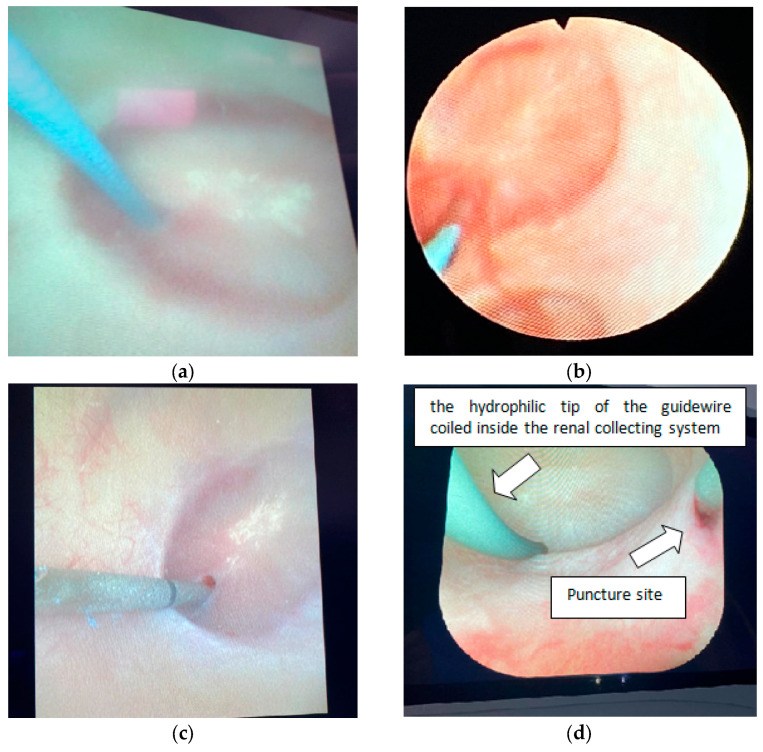

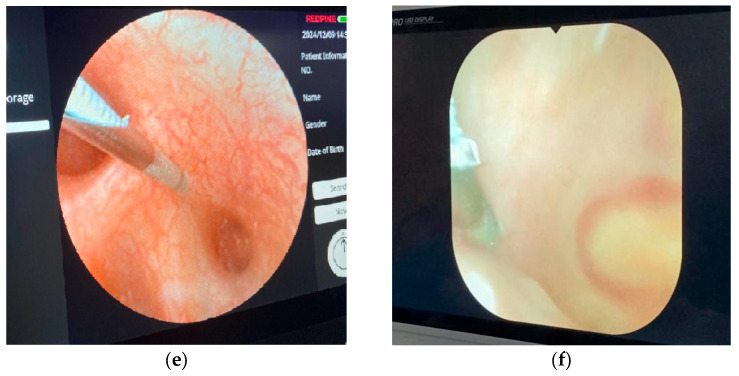
Endoscopic views of renal accesses using flexible ureteroscopy: (**a**–**c**) transpapillary accesses; (**d**–**f**) nonpapillary accesses. (Various disposable flexible ureteroscopes from different manufacturers were utilized during the procedures. Additionally, the Storz Flex-X2^®^ (Karl Storz, Tuttlingen, Germany) was frequently employed for flexible ureteroscopy).

Freehand ultrasound-guided (F-UG) puncture: The surgeon (BE), experienced in F-UG percutaneous renal access, performed all punctures in the ultrasound-guided group using a freehand technique without a needle guide [14]. Following the instillation of saline through the ureteral access sheath to dilate the collecting system, the surgeon evaluated the kidney using ultrasonography. After assessing the anatomy with the ultrasound probe in the longitudinal position, the surgeon selected the desired calyx and aimed to achieve a transpapillary renal puncture [15]. Once the surgeon confirmed the adequacy of the access, a guidewire was advanced into the collecting system through the percutaneous access needle. Subsequently, a flexible ureteroscope was introduced through the ureteral access sheath to assess whether the puncture was transpapillary or nonpapillary (Figure 2).

Fluoroscopy-guided (FG) puncture: The surgeon (FB), experienced in FG percutaneous renal access, performed all punctures in the fluoroscopy-guided group using the biplanar technique [16]. Following the injection of a contrast medium through the ureteral access sheath to perform retrograde pyelography and visualize the collecting system, the surgeon evaluated the renal anatomy under fluoroscopy. After assessing the anatomy, the surgeon selected the desired calyx and aimed to achieve a transpapillary renal puncture. Once the surgeon confirmed the adequacy of the access, a guidewire was advanced into the collecting system through the percutaneous access needle. Subsequently, a flexible ureteroscope was introduced through the ureteral access sheath to assess whether the puncture was transpapillary or nonpapillary (Figure 2).

After renal access, tract dilation was performed with Amplatz serial dilators (Geotek^®^, Ankara, Türkiye) under fluoroscopic guidance in all patients, followed by the placement of a 24 Fr Amplatz sheath. The remainder of the surgical procedure, including stone fragmentation with a holmium laser or/and pneumatic lithotripter and retrieval, was performed by the same surgeon (FB) for all patients to ensure procedural consistency. At the end of the procedures, the placement of a ureteral catheter (Double J stent, Plastimed^®^, Ankara, Türkiye) and nephrostomy were performed based on the surgeon’s preference.

### 2.6. Clinical and Perioperative Data Collection and Follow-Up

Demographic data such as age, gender, body mass index (BMI), history of previous surgery, additional comorbidities, and serum biochemistry results were recorded. Stone-related parameters, including side, localization, size, volume, opacity, and Hounsfield unit (HU), as well as surgical information such as operation time, fluoroscopy times, puncture time, renal access (papillary or nonpapillary), complications (the Clavien–Dindo classification) [17], hospitalization time, and stone-free (SF) rate, were also documented. In addition, stone complexity was assessed using the Guy’s Stone Score (GSS), a validated scoring system for evaluating stone burden and anatomical factors relevant to percutaneous nephrolithotomy [18].

Stone-free status was evaluated using NCCT in the first postoperative month. The “stone-free” status was defined as the complete absence of any stone fragments. “Clinically insignificant residual fragments (CIRFs)” status was defined as asymptomatic stone fragments (non-obstructive and not causing infection) smaller than 4 mm. “Failure” status was defined as stone fragments larger than 4 mm or symptomatic stone fragments (obstructive or causing infection) smaller than 4 mm.

### 2.7. Statistical Analysis

All statistical analyses were performed using SPSS version 21.0 (IBM Corp., Armonk, NY, USA). The normality of continuous variables was assessed using the Shapiro–Wilk test. Variables that followed a normal distribution were analyzed using the Student’s *t*-test, while variables that did not follow a normal distribution were analyzed using the Mann–Whitney U test. Categorical variables were compared using the Chi-square test. When the expected frequency in any cell was less than 5, Fisher’s exact test was used to ensure the accuracy of the results. A *p*-value of less than 0.05 was considered statistically significant.

A post hoc power analysis was performed using the G Power program to evaluate the statistical power of our comparison between F-UG puncture and FG puncture in achieving transpapillary access. Based on the observed transpapillary puncture rates in Group 1 (n = 23) and Group 2 (n = 20), the analysis yielded an achieved power (1-β) of 90.11% with a significance level (α) of 0.05, confirming an adequate sample size for detecting the observed difference.

## 3. Results

### 3.1. Demographic and Clinical Characteristics

Table 1 summarizes the demographic and clinical characteristics of the patients who underwent PNL using FG access and F-UG access (without a needle guide).

### 3.2. Stone Characteristics

Table 2 presents the stone characteristics of the patients in both groups.

### 3.3. Perioperative and Postoperative Outcomes

Table 3 summarizes the intraoperative and postoperative parameters:

- The targeted calyx for access differed significantly between groups (***p*: 0.043**), with more inferior calyx access in the FG group.
- No fluoroscopy was used for renal access in the F-UG group.
- The average puncture time was significantly shorter in the FG group (***p* < 0.001**).
- The renal access type showed a statistically significant difference, with the FG group having more transpapillary access (***p*: 0.003**).
- The total fluoroscopy time was significantly lower in the F-UG group (***p* < 0.001**).
- Complication rates, classified using the Clavien–Dindo system, showed no significant difference (*p*: 0.092). The FG group had no complications. The F-UG group had two Clavien Grade 2 (bleeding required blood transfusion) and one Clavien Grade 4a (admission to the intensive care unit due to urosepsis and administration of appropriate intravenous antibiotic therapy) complications.

**Table 3:** Perioperative and postoperative parameters and outcomes.

				FG Access (n: 23)	F-UG Access (n: 20)	*p*-Value
Targeted calyx for access	Inferior calyx			14 (60.9%)	6 (30.0%)	0.043 **
	Middle calyx			9 (39.1%)	14 (70.0%)	
Lithotripsy method	Pneumatic			2 (8.7%)	4 (20.0%)	0.446 ****
	Laser			18 (78.3%)	15 (75.0%)	
	Pneumatic + laser			3 (13.0%)	1 (5.0%)	
D/J stent	Yes			23 (100.0%)	19 (95.0%)	0.465 ****
	No			0 (0.0%)	1 (5.0%)	
Nephrostomy tube	Yes			1 (4.3%)	3 (15.0%)	0.323 ****
	No			22 (95.7%)	17 (85.0%)	
Number of puncture attempts for access (mean ± st. deviation/median/min-max)				2.5 ± 1.7	1.8 ± 0.8	0.177 ***
				2.0	2.0	
				1.0–8.0	1.0–4.0	
Fluoroscopy time for percutaneous renal access (second) (mean ± st. deviation/min-max)				15.3 ± 12.7	0.0	
				5.0–60.0		
Average puncture time (minute) (mean ± st. deviation/median/min-max)				2.51 ± 2.34	5.68 ± 3.17	<0.001 ***
				2.00	5.50	
				0.50–10.00	0.75–14.00	
**Renal access**		**Transpapillary**		22 (95.7%)	11 (55.0%)	**0.003** ****
		**Nonpapillary**		1 (4.3%)	9 (45.0%)	
Total fluoroscopy time (second) (mean ± st. deviation/median/min-max)				23.3 ± 12.7	7.8 ± 4.8	<0.001 ***
				20.0	7.0	
				10.0–66.0	2.0–20.0	
Operation time (minute) (mean ± st. deviation/median/min-max)				79.6 ± 28.0	72.2 ± 13.9	0.546 ***
				70.0	70.0	
				45.0–160.0	50.0–95.0	
Complications (Clavien–Dindo classification)			No	23 (100.0%)	17 (85.0%)	0.092 ****
			Grade 2	0 (0.0%)	2 (10.0%)	
			Grade 4a	0 (0.0%)	1 (5.0%)	
Hematocrit drop (mean ± st. deviation/median/min-max)				2.92 ± 1.44	4.46 ± 3.20	0.428 ***
				2.90	3.20	
				0.00–6.20	0.00–14.50	
Blood transfusion	Yes			0 (0.0%)	2 (10.0%)	0.210 ****
	No			23 (100.0%)	18 (90.0%)	
Hospitalization time (day) (mean ± st. deviation/median/min-max)				1.5 ± 0.5	2.1 ± 1.4	0.258 ***
				2.0	2.0	
				1.0–2.0	1.0–7.0	
1st-month stone-free rate	Stone-free			21 (91.4%)	17 (85.0%)	0.790 ****
	CIRFs			1 (4.3%)	2 (10.0%)	
	Failure			1 (4.3%)	1 (5.0%)	

** Chi-square test. *** Mann–Whitney U test. **** Fisher’s exact test. Abbreviations—FG: fluoroscopy-guided; F-UG: freehand ultrasound-guided; D/J: double J; CIRFs: clinically insignificant residual fragments. Note: Bold values indicate statistical significance (*p* < 0.05) and emphasize the primary endpoint of the study, renal access.

## 4. Discussion

One of the key initial steps in performing PNL is achieving access to the renal collecting system via a suitable calyx. Imaging guidance, most commonly fluoroscopy or ultrasound, is typically utilized to ensure accurate and safe access [19]. Since the early 1990s, the generally accepted technique has been transpapillary access through the fornix of the renal papilla after Sampaio’s studies [4,5]. However, following the publications of Liatsikos et al. [6,7], the distinction between papillary and nonpapillary puncture has gained attention and sparked ongoing debate in the literature. Despite this, there is a lack of data regarding the true proportion of cases in which genuine transpapillary access is achieved, regardless of the intended technique. It should also be acknowledged that even experienced endourologists aiming for a papillary puncture may not achieve technically perfect transpapillary access in every single case. Consequently, a considerable portion of the reported outcomes in the literature are likely based on punctures that were not genuinely transpapillary [20]. Among the various available methods, direct endoscopic observation of the needle entering through the papillary fornix is considered the most reliable technique to confirm anatomically accurate transpapillary access. Although this approach has been known and utilized for many years, it has not been widely adopted in routine clinical practice. Moreover, even in studies that employed fluoroscopy with ureteroscopic observation of the needle during percutaneous access, such as the one conducted by Khan et al., in which one of the eleven patients required transfusion due to bleeding and the procedure was terminated, there was no explicit mention of whether the punctures were transpapillary or not [21]. Despite the use of advanced visualization techniques, the anatomical character of the access site (transpapillary versus nonpapillary) was not specified, further highlighting the absence of concrete data on this issue in the current literature. This underscores the need for more definitive data on the anatomical nature of percutaneous access in current clinical practice. In this context, the primary endpoint of our prospective, comparative pilot study was to compare two renal access techniques routinely employed in our institution, F-UG puncture and FG puncture, concerning the rate of anatomically accurate transpapillary access. A comprehensive review of the literature revealed no prior studies that directly compare these two methods in terms of their ability to achieve transpapillary puncture. Therefore, to the best of our knowledge, this study represents the first investigation in the field to specifically address this technical aspect, aiming to contribute novel insight to an area that remains underexplored. As a secondary endpoint, perioperative and postoperative parameters were analyzed to assess the efficiency and safety of each technique. Particular attention was given to complications, especially bleeding and the need for transfusion, which are among the most critical safety concerns in percutaneous nephrolithotomy. It is also important to emphasize that in this study, once access was achieved and considered by the surgeon to be appropriate and safe for proceeding with the operation, regardless of whether it was papillary or nonpapillary in endoscopic view, it was not altered or repositioned. This approach allowed for a consistent and objective evaluation of the impact of the initial puncture on clinical outcomes, independent of any subsequent procedural modifications.

In this prospective, comparative pilot study, the primary endpoint was to compare the effectiveness of F-UG versus FG puncture techniques in achieving anatomically accurate transpapillary access, as verified through direct endoscopic visualization. Our findings demonstrated that F-UG puncture resulted in a significantly lower rate of transpapillary access compared to the FG approach (55.0% vs. 95.7%, *p* = 0.003). To the best of our knowledge, this is the first prospective study in the literature to directly compare these two commonly used imaging modalities with endoscopic confirmation of the anatomical character of the access site. Even in studies attempting to verify the nature of renal access, confirmation has typically relied on secondary imaging modalities (fluoroscopy or ultrasound) rather than direct endoscopic visualization, leaving uncertainty regarding the true anatomical character of the puncture [11,12]. Likewise, in the randomized controlled trial that significantly contributed to the transpapillary versus nonpapillary puncture debate, the verification of the access site was performed using the same imaging modality (fluoroscopy) that was initially used to obtain the access, potentially introducing confirmation bias and limiting the anatomical precision of the assessment (i.e., the actual proportion of papillary accesses that represent true transpapillary access remains unclear) [13]. Although limited, one relevant study by Basiri et al. evaluated ultrasound-guided access (not freehand technique, with a needle guide). It defined a “proper entry site” as puncture through the surface of the renal papilla, rather than from the lateral walls of the calyx or other locations. The same study also defined an “appropriate direction” as a trajectory leading toward the renal pelvis. According to these criteria, successful access (including proper entry site and appropriate direction) was achieved in 72% of cases [11]. While this provides a useful reference point for evaluating the efficacy of ultrasound-guided access, it is important to note that the study relied entirely on imaging-based evaluation (verification with fluoroscopy) and did not utilize endoscopic confirmation. In this context, the current study offers a more objective assessment of access accuracy and contributes to a clearer understanding of the true anatomical outcome of commonly employed puncture techniques. It is also important to acknowledge that differences in ultrasound-guided access outcomes may, in part, be attributed to variations in technique. In the study by Basiri et al., a needle guide was utilized to facilitate accurate needle placement, allowing real-time visualization of the intended path via an electronic dotted line. In contrast, our study employed a freehand approach without a needle guide. According to Desai, ultrasound-guided access without a needle guide is considered suboptimal, as it lacks precise control over needle depth and alignment, which may compromise puncture accuracy, especially in systems with minimal hydronephrosis [14]. This technical variation may partly explain the lower rate of true transpapillary access observed in our ultrasound-guided group, despite similar procedural goals. Additional insight into the anatomical accuracy of ultrasound-guided access comes from a study evaluating ureteroscope-assisted ultrasound-guided puncture, in which flexible ureteroscopy was used intraoperatively to confirm the puncture site. In cases where the access was deemed anatomically inappropriate (such as nonpapillary entry), new punctures were performed until correct calyceal access was achieved. Although the exact frequency of such adjustments was not reported, the study demonstrated that nonpapillary accesses can occur even under ultrasound guidance and may require correction upon visual confirmation [22]. In this regard, our study not only demonstrates the comparative effectiveness of two commonly used access techniques but also provides a scientific foundation for future investigations aiming to standardize renal access methods using direct endoscopic verification. The methodological framework employed in our study may serve as a basis for further comparative studies that could help resolve the ongoing debate regarding transpapillary versus nonpapillary access. Moreover, objective studies are warranted to subclassify nonpapillary accesses—such as transpapillary, peripapillary, infundibular, or pelvic—and to evaluate their association with clinical outcomes and complication rates (especially bleeding). Such efforts could contribute to a more refined understanding of the anatomical and functional implications of different puncture trajectories in percutaneous nephrolithotomy.

Following the evaluation of the primary endpoint, the analysis was extended to include secondary outcomes, focusing on perioperative and postoperative parameters to further assess the safety and efficiency of each access technique in clinical practice. In our study, the mean number of puncture attempts required to achieve renal access was higher in the F-UG group compared to the FG group (1.8 ± 0.8 vs. 2.5 ± 1.7), although this difference did not reach statistical significance (*p* = 0.177). This result aligns closely with the findings of Jagtap et al., who conducted a prospective randomized trial involving trainee urologists and reported no significant difference in puncture attempts between ultrasound-guided and fluoroscopy-guided access (1.7 ± 0.9 vs. 1.6 ± 0.8; *p* = 0.33) [23]. Interestingly, while Agarwal et al. reported a significantly higher number of puncture attempts in the fluoroscopy-guided group (3.3 vs. 1.5; *p* < 0.01), Birowo et al. found the opposite, with significantly more attempts required in the ultrasound-guided group compared to both prone and supine fluoroscopy-guided access (2.0 ± 1.7 vs. 1.3 ± 0.5 and 1.1 ± 0.2, respectively; *p* < 0.01) [24,25]. These contradictory findings highlight the lack of consensus in the current literature regarding which modality offers greater efficiency in terms of puncture attempts, suggesting that operator experience, technique (freehand vs. needle-guided), and patient-related factors may significantly influence outcomes.

In our study, the choice of targeted calyx for renal access differed significantly between the two groups (*p* = 0.043). While the inferior calyx was predominantly preferred in the FG group (60.9%), the middle calyx was more frequently selected in the F-UG group (70.0%). This calyceal selection pattern is in line with findings from previous studies. For instance, in the multicenter CROES Global PCNL Study analyzed by Andonian et al., middle calyx access was significantly more common in the ultrasound-guided group, whereas lower pole access was favored in the fluoroscopy-guided group (middle calyx access: 39.2% vs. 11.1%, *p* < 0.001; lower pole access: 44.8% vs. 68.1%, *p* < 0.001) [26]. Likewise, Birowo et al. reported that in their prospective series of supine PCNL cases, mid-pole punctures were significantly more frequent in the ultrasound group compared to both supine and prone fluoroscopy-guided groups (42.5% vs. 5.0% and 10.0%, respectively) [25]. This shift in calyceal preference may be explained by the inherent differences between imaging modalities. Ultrasound provides real-time spatial orientation and easier identification of more anterior and superficial calyces—such as the middle calyx—especially in the supine position.

As expected, the total fluoroscopy time was significantly higher in the FG group compared to the F-UG group. The mean total fluoroscopy time was 23.3 ± 12.7 s in the fluoroscopy group versus 7.8 ± 4.8 s in the ultrasound-guided group (*p* < 0.001). This result is consistent with the fundamental advantage of ultrasound guidance in minimizing radiation exposure. Similar findings have been reported in the literature. Basiri et al., Agarwal et al., and Jagtap et al. all demonstrated a significantly higher mean fluoroscopy time in the fluoroscopy-guided access groups of their studies [23,24,27]. Repeated exposure to ionizing radiation may pose long-term health risks for both patients and operating room personnel [28]. Therefore, even though the radiation dose during PNL typically remains within acceptable safety thresholds, minimizing exposure is a priority in modern endourological practice. In this context, ultrasound-guided access offers a distinct advantage by significantly reducing or eliminating the need for fluoroscopy, thereby enhancing intraoperative safety.

In our study, both the operation time and hospitalization time were statistically comparable between the F-UG and FG groups, suggesting that the choice of imaging modality did not significantly influence the overall surgical workflow or postoperative recovery. These results are in line with two recent meta-analyses published in 2023 and 2024, both of which found no significant differences between ultrasound-guided and fluoroscopy-guided PNL in terms of operative time and length of hospital stay across large patient populations [29,30]. In our cohort, the only parameter that showed a statistically significant difference between the two groups was the average puncture time, which was longer in the F-UG group. This finding is in line with both recent meta-analyses by Bahri et al. (2023) and Du et al. (2024), which reported that access time was significantly shorter in the fluoroscopy-guided PNL group [29,30]. These results suggest that although ultrasound guidance eliminates radiation exposure, it may require a steeper learning curve and a longer time to achieve optimal access. Additionally, in our study, the longer puncture time observed in the ultrasound-guided group may be attributed to the use of a freehand technique without a needle guide, which requires the operator to simultaneously interpret the ultrasound image and control needle advancement, potentially increasing the technical complexity and duration of access.

The stone-free rate (SFR) at the 1st-month follow-up was 91.4% in the fluoroscopy-guided group and 85.0% in the ultrasound-guided group. Clinically insignificant residual fragments (CIRFs) were detected in 4.3% and 10.0% of patients, respectively, while failure was noted in 4.3% and 5.0%. The difference in the stone-free rates between the two groups was not statistically significant (*p* = 0.790). These results are consistent with the majority of the literature, including four of five meta-analyses, which reported no significant difference between the two techniques [29,30,31,32]. Only Wang et al.’s 2015 meta-analysis showed a statistically significant advantage for ultrasonographic access (SFR: 86.2% vs. 81.9%; *p* = 0.03) [33]. Therefore, our findings align with the broader literature, supporting that both access methods achieve comparable stone-free rates in PNL.

Although the complication rate appeared higher in the F-UG group, the difference was not statistically significant (*p* = 0.092). In the FG group, no complications were observed (0/23; 0%). In contrast, in the F-UG group, two patients (2/20; 10.0%) experienced Clavien Grade 2 complications (bleeding required blood transfusion), and one patient (1/20; 5.0%) experienced a Clavien Grade 4a complication (admission to the intensive care unit due to urosepsis and administration of appropriate intravenous antibiotic therapy). Similarly, the mean hematocrit drop was higher in the F-UG group (4.46 ± 3.20 vs. 2.92 ± 1.44), and blood transfusions were required in two patients (10.0%) in this group, whereas none of the patients in the FG group required transfusion (0%). However, these differences were not statistically significant. Interestingly, although two transfusions were observed in the F-UG group, the underlying causes differed, providing valuable insight into the potential mechanisms of bleeding. One patient who underwent nonpapillary access experienced persistent bleeding that began immediately after tract dilation, suggesting injury to renal parenchymal vessels due to an anatomically unfavorable access point. The second transfusion occurred in a patient who had transpapillary access, but the puncture was directed through the middle calyx toward a pelvic stone. However, in contrast to the ideal “appropriate direction (angle)” (defined as a trajectory leading toward the renal pelvis by Basiri et al. [11]), the needle did not follow a straight path along the infundibulum. As a result, during rigid nephroscopy, while attempting to reach the stone in the renal pelvis, the neck of the calyx (infundibulum) was inadvertently torn. This led to intraoperative bleeding. At the end of the procedure, antegrade pyelography revealed extravasation of contrast into the renal vein with subsequent passage into the inferior vena cava, confirming a segmental venous injury. These two cases underscore that both access type (papillary/nonpapillary) and access direction (angle) may play critical roles in the risk of bleeding. Even a correctly placed transpapillary puncture may lead to vascular injury if not aligned properly with the collecting system anatomy, especially when approaching pelvic stones through an inappropriate tract direction. This highlights the importance of not only targeting the correct calyx but also ensuring an optimal angle and trajectory to minimize complications. Contrary to our findings, in the large CROES PNL Global Study by Andonian et al., the rate of bleeding and blood transfusion was significantly higher in the fluoroscopy-guided group (13.8% vs. 6.0% and 11.1% vs. 3.8%, respectively; both *p* < 0.001) compared to the ultrasound-guided group [26]. Similarly, in a randomized clinical trial conducted by Falahatkar et al., where both groups underwent complete supine PCNL, all four cases that required blood transfusion were observed exclusively in the fluoroscopy group, while no transfusions were needed in the ultrasound-guided group [34]. These contrasting results may be attributed to differences in patient characteristics, access techniques, sheath sizes, and surgeon experience across studies. Nonetheless, a recent systematic review and meta-analysis published in 2024 by Du et al., including 21 randomized controlled trials, offered a broader perspective. Their pooled analysis of nine studies involving 1362 patients found no significant difference in blood transfusion rates between ultrasound-guided and fluoroscopy-guided PNL. Likewise, an analysis of 13 studies including 1591 patients showed no statistically significant difference in hemoglobin drop [30]. These comprehensive findings indicate that, while some individual studies report more bleeding events in the fluoroscopy group, aggregated data do not support a consistent statistically significant difference between the two techniques. From the perspective of our study, although the post hoc power analysis confirmed sufficient statistical power for the primary endpoint, which was successful transpapillary access, the relatively limited sample size constitutes a major limitation of this pilot study. Consequently, the study may not have had adequate statistical power to detect significant differences in secondary endpoints such as complication rates and the need for blood transfusion. Therefore, the lack of statistically significant findings in these outcomes should be interpreted with caution. Interestingly, no Grade 1 complications were observed in our cohort. According to the Clavien–Dindo classification, Grade 1 complications are defined as any deviation from the normal postoperative course that does not require pharmacological treatment or surgical, endoscopic, or radiological interventions. These typically include mild, self-limited events such as transient hematuria, low-grade fever, mild pain not requiring opioids, nausea, or irritative lower urinary tract symptoms (e.g., dysuria, urgency). Such events are frequently underreported in studies, especially when they do not necessitate specific medical management. In our study, this absence may reflect both the relatively small sample size and the tendency to overlook or omit minor self-limiting postoperative symptoms in routine documentation when they resolve spontaneously and have no clinical consequence. 

This study possesses several key strengths that contribute meaningfully to the field of endourology. To our knowledge, it is the first prospective study to compare freehand ultrasound-guided and fluoroscopy-guided punctures with confirmation of true transpapillary access via direct endoscopic visualization, which is considered the most reliable method for anatomical verification. The study’s methodological rigor, including the use of strict inclusion and exclusion criteria, ensured a homogeneous and well-defined patient population, enhancing internal validity. Importantly, once initial access was achieved, it was not modified, allowing for an objective assessment of its direct impact on surgical outcomes. The study also provides a comprehensive perioperative evaluation, including parameters such as puncture characteristics, radiation exposure, complications, and stone-free status. Furthermore, the direct endoscopic confirmation protocol adopted here may serve as a standardized methodological framework for future studies aiming to compare transpapillary and nonpapillary access techniques. These features collectively provide a solid scientific foundation for further research aiming to optimize and standardize renal access in percutaneous nephrolithotomy.

This study has several limitations that should be acknowledged. First, although post hoc power analysis confirmed sufficient statistical power for the primary endpoint of transpapillary access, the relatively small sample size may have limited the ability to detect statistically significant differences in secondary outcomes such as complication rates and blood transfusion rates. Therefore, the lack of significance in these parameters should be interpreted with caution. Second, ultrasound-guided punctures in this study were performed using a freehand technique without a needle guide. This approach may differ technically and ergonomically from ultrasound-guided procedures that utilize a needle guide for real-time trajectory assistance. As such, the findings related to freehand ultrasound-guided access in this study may not be directly generalizable to ultrasound-guided techniques employing a needle holder or bracket system. Although both surgeons were experienced in PNL, their level of experience with the specific access technique used (FG vs. F-UG) was not identical, which may have influenced certain perioperative outcomes such as puncture time and accuracy. Stone complexity was evaluated using the Guy’s Stone Score, which, while informative, may not fully capture intraoperative variability or subtle anatomical differences that could influence renal access (transpapillary/nonpapillary). Moreover, endoscopic confirmation was utilized solely to verify the anatomical location of the access site (transpapillary vs. nonpapillary) and did not involve a systematic evaluation of potential tract-related injuries, which may limit insight into the relative safety of each technique. Finally, the study was conducted in a single-center setting by two surgeons using distinct imaging modalities, which may limit the generalizability of the results to broader clinical practice.

## 5. Conclusions

The results of this prospective, comparative pilot study suggest that fluoroscopy-guided puncture may be more effective in achieving anatomically accurate transpapillary access, while freehand ultrasound-guided access offers the benefit of significantly reduced radiation exposure. Complication rates, including bleeding and transfusion, were similar between the two groups, although the limited sample size may have precluded the detection of significant differences. As this is a pilot study with a relatively small cohort, the findings should be interpreted with caution and not be overgeneralized beyond the observed data. Further studies with larger sample sizes are warranted to more objectively evaluate safety outcomes, particularly concerning bleeding complications.

## Figures and Tables

**Table 1:** Demographics of the patients.

		FG Access (n: 23)	F-UG Access (n: 20)	*p*-Value
Age (mean ± st. deviation/median/min-max)		47.6 ± 15.0	46.5 ± 13.3	0.802 *
		49.0	45.5	
		22.0–81.0	24.0–79.0	
Gender	Male	18 (78.3%)	10 (50.0%)	0.052 **
	Female	5 (21.7%)	10 (50.0%)	
BMI (body mass index) (mean ± st. deviation/median/min-max)		26.49 ± 4.26	27.19 ± 2.72	0.210 ***
		26.10	27.35	
		19.50–37.90	22.00–33.10	
Comorbidities (diabetes, hypertension, etc.)	Yes	12 (52.2%)	6 (30%)	0.142 **
	No	11 (47.8%)	14 (70%)	
Previous SWL/surgery	SWL	4	5	
	URS	3	2	
	RIRS	0	1	
	PNL	4	0	
	Open surgery	1	0	
	Lap. surgery	1	0	

* Student’s *t*-test. ** Chi-square test. *** Mann–Whitney U test. Abbreviations—FG: fluoroscopy-guided; F-UG: freehand ultrasound-guided; BMI: body mass index; SWL: shockwave lithotripsy; URS: ureteroscopy; RIRS: retrograde intrarenal surgery; PNL: percutaneous nephrolithotomy.

**Table 2:** Stone characteristics of the patients.

		FG Access (n: 23)	F-UG Access (n: 20)	*p*-Value
Stone side	Right	16 (69.6%)	10 (50.0%)	0.191 **
	Left	7 (30.4%)	10 (50.0%)	
Stone opacity	Opaque	18 (78.3%)	17 (85.0%)	0.704 ****
	Semi-opaque	5 (21.7%)	3 (15.0%)	
	Non-opaque	0 (0.0%)	0 (0.0%)	
Hydronephrosis	No	7 (30.4%)	3 (15.0%)	0.361 ****
	Grade 1	7 (30.4%)	10 (50.0%)	
	Grade 2	9 (39.2%)	7 (35.0%)	
Stone size (mm) (mean ± st. deviation/median/min-max)		23.5 ± 5.0	23.8 ± 5.1	0.841 ***
		22.0	22.0	
		18.0–39.0	19.0–38.0	
Stone volume (mm^3^) (length × width × depth × π/6) (mean ± st. deviation/median/min-max)		3413 ± 2308	3495 ± 2039	0.534 ***
		2787	2966	
		1313–10128	1313–10160	
Hounsfield unit (mean ± st. deviation/median/min-max)		1117 ± 251	1019 ± 250	0.208 *
		1135	935	
		442–1505	465–1447	
Stone localization	Pelvis	17 (73.9%)	18 (90.0%)	0.250 ****
	Lower calyx	6 (26.1%)	2 (10.0%)	
The Guy’s Stone Score	Grade 1	23 (100%)	20 (100%)	⸸

* Student’s *t*-test. ** Chi-square test. *** Mann–Whitney U test. **** Fisher’s exact test. ⸸ All patients in both groups were classified as Guy’s Stone Score Grade 1. Since there was no variation in stone complexity, statistical comparison was not applicable. Abbreviations—FG: fluoroscopy-guided; F-UG: freehand ultrasound-guided.

## Data Availability

The data supporting the findings of this study are available from the corresponding author upon reasonable request. Due to privacy and ethical restrictions, the dataset is not publicly accessible.

## Abbreviations

The following abbreviations are used in this manuscript:

PNL	Percutaneous nephrolithotomy
F-UG	Freehand ultrasound-guided
FG	Fluoroscopy-guided
NCCT	Non-Contrast-Enhanced Computed Tomography
BMI	Body mass index
HU	Hounsfield unit
SF	Stone-free
CIRFs	Clinically insignificant residual fragments
